# Selection of Reserves for Woodland Caribou Using an Optimization Approach

**DOI:** 10.1371/journal.pone.0031672

**Published:** 2012-02-20

**Authors:** Richard R. Schneider, Grant Hauer, Kimberly Dawe, Wiktor Adamowicz, Stan Boutin

**Affiliations:** 1 Department of Biological Sciences, University of Alberta, Edmonton, Alberta, Canada; 2 Department of Resource Economics and Environmental Sociology, University of Alberta, Edmonton, Alberta, Canada; Umea University, Sweden

## Abstract

Habitat protection has been identified as an important strategy for the conservation of woodland caribou (*Rangifer tarandus*). However, because of the economic opportunity costs associated with protection it is unlikely that all caribou ranges can be protected in their entirety. We used an optimization approach to identify reserve designs for caribou in Alberta, Canada, across a range of potential protection targets. Our designs minimized costs as well as three demographic risk factors: current industrial footprint, presence of white-tailed deer (*Odocoileus virginianus*), and climate change. We found that, using optimization, 60% of current caribou range can be protected (including 17% in existing parks) while maintaining access to over 98% of the value of resources on public lands. The trade-off between minimizing cost and minimizing demographic risk factors was minimal because the spatial distributions of cost and risk were similar. The prospects for protection are much reduced if protection is directed towards the herds that are most at risk of near-term extirpation.

## Introduction

Woodland caribou herds are declining across much of their range in Canada, prompting the development of recovery strategies at the federal and provincial levels [1]. The protection of caribou habitat has been identified as an important component of recovery efforts, given the underlying role of anthropogenic disturbances in the decline of caribou populations [2]–[5]. The implementation of protection is challenging, however. Caribou ranges are typically thousands of square kilometres in size, meaning that a prohibition on industrial activities may carry a large economic opportunity cost (i.e., the value of foregone resource development opportunities).

Because of the trade-off between habitat protection and resource development it is unlikely that all ranges can be protected in their entirety [6]. A decision must therefore be made as to which areas will be protected, and which will not. One approach is to place a priority on herds that are at immediate risk of extirpation. Managers are likely to utilize this approach if the loss of even one herd is considered an unacceptable outcome. In this study we explore an alternative approach in which protection is allocated strategically, at the township scale (∼9500 ha), with the aim of maximizing overall conservation gains given economic constraints [7], [8]. This work builds on an earlier study that documented differences among caribou herds in Alberta with respect to the cost of recovery efforts and various measures of long-term viability [6].

**Figure 1:**
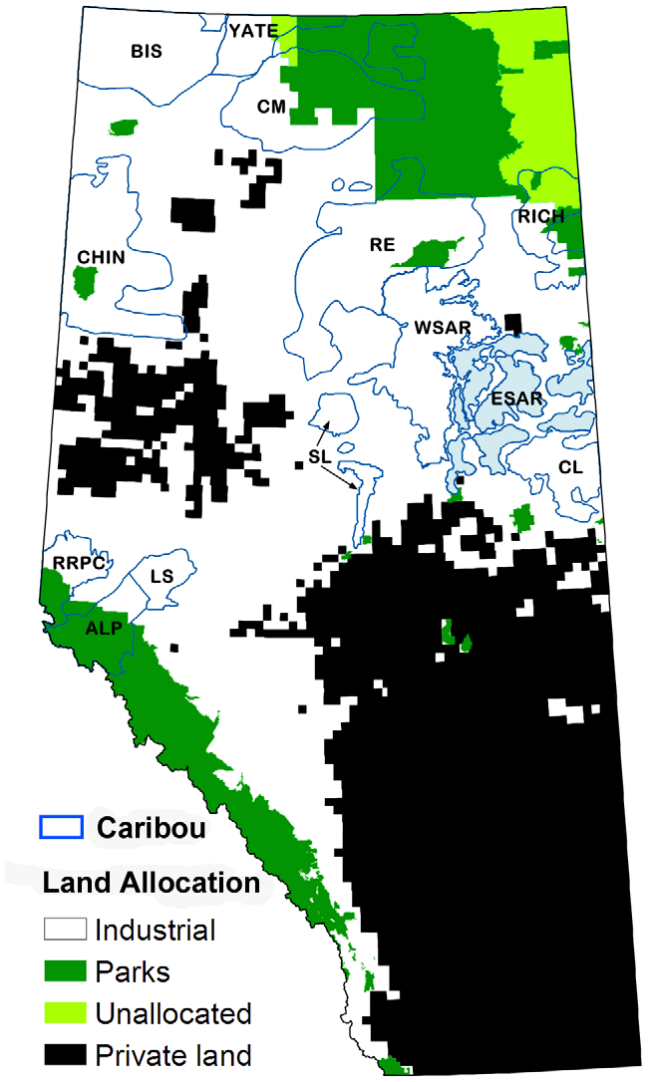
Study area for the Marxan modeling. Major land-use zones and the location of caribou ranges are shown. The land allocation labelled “Industrial” refers to oil and gas and forestry tenures on public lands. Caribou ranges are individually labelled (see Table 1) and the ESAR range is shaded for clarity.

Various techniques have been developed in recent years for optimizing the allocation of conservation resources [9]–[11]. These techniques involve the use of algorithms that provide optimal solutions to mathematically defined expressions of management options and objectives. We apply these techniques to the selection of reserves for woodland caribou in Alberta, Canada, using the Marxan conservation planning software [12]. There are approximately 3000 caribou in the province, split into 13 main herds (Fig. 1). All herds but one have experienced negative population growth in recent years (Table 1) and nine of the 13 herds will decline to less than ten animals over the next 35 years if current demographic trends continue [6].

**Table 1:** Population data for caribou herds in Alberta.

Herd	Label	Range size (km^2^)	Growth rate^a^
A La Peche	ALP	6,615	0.93
Athabasca River East	ESAR	13,154	0.86
Athabasca River West	WSAR	15,707	0.94
Bistcho	BIS	14,358	0.99
Caribou Mountains	CM	20,659	0.89
Chinchaga	CHIN	17,644	0.88
Cold Lake	CL	6,726	0.78
Little Smoky	LS	3,084	0.89
Red Earth	RE	24,702	0.91
Redrock - Prairie Creek	RRPC	4,829	0.93
Richardson	RICH	7,074	0.97
Slave Lake	SL	3,621	0.95
Yates	YATE	5,223	1.05

a Geometric mean of annual population growth rate from 2004–2009, based on survival and recruitment data collected by the Alberta Caribou Committee. Only two years of data were available for YATE and RICH, and data for LS are for the 5-year period before wolf control was initiated in 2007.

Our objective is to identify reserve designs across a range of protection targets that minimize opportunity costs as well as three demographic risk factors: current industrial footprint, presence of white-tailed deer, and climate change (see below). In practical terms, we seek to prioritize caribou range at the township scale with respect to its ability to contribute to the long-term viability of caribou in the province. This provides the basis for strategic decision making, though we note that land managers will need to consider additional factors such as minimum reserve size when designating reserves.

**Figure 2:**
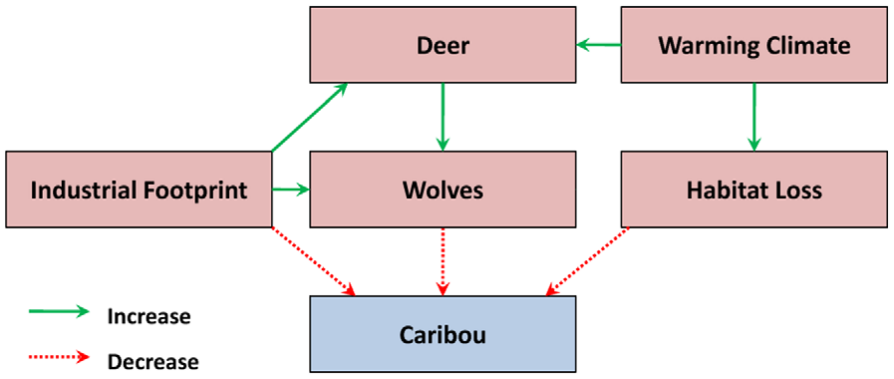
Impact hypothesis diagram for selected factors affecting caribou viability.

We utilized three different measures of demographic risk in our model in order to be as comprehensive as possible in assessing prospects for long-term caribou viability. Although various interrelationships exist among these factors, their cumulative influence cannot be captured through any one factor (Fig. 2). We did not include predation risk from wolves (*Canis lupus*) in our model because spatially defined estimates of wolf density are not available for our study area. But we note that wolves and caribou have long coexisted, so there is no reason to believe that wolf predation would lead to the decline of caribou were it not for the other factors in our model.

Industrial disturbances influence caribou indirectly through an increase in the rate of predation, primarily by wolves. The leading hypothesis is that anthropogenic alterations in forest structure lead to: (1) an increase in the density of white-tailed deer and moose (*Alces alces*), (2) an increase in the density of wolves, (3) an increased rate of encounter between wolves and caribou, and (4) increased wolf hunting efficiency [2], [5], [13]–[16]. Caribou tend to avoid anthropogenic features, so it is possible that industrial disturbances may also have direct effects on caribou demographics [13].

We include white-tailed deer as a separate risk factor because industrial disturbance is not the only cause of the expansion in deer range observed in recent years [16]–[18]. Winter severity, which has been affected by global warming, and proximity to agricultural lands, which continue to expand in northwestern Alberta, are two additional factors that are having an effect [18]–[20].

In the near-term the influence of climate change is likely to be indirect, through the positive effect that reduced winter severity and lengthened growing season will have on deer populations [18], [21], [22]. Over the longer term, climate change is projected to result in the loss of suitable habitat within some ranges. By 2050, some caribou range will experience the climate that is currently found in parkland regions, eventually leading to a transformation in vegetation [23].

Our optimization model also takes the economic opportunity cost of protection into account. By seeking the most cost effective design we ensure that whatever societal cost limitations are imposed, the amount of protection achieved will be the greatest possible [9], [10], [24].

The final element of our analysis considers the protection needs of other species. The industrial disturbances threatening caribou in Alberta also affect other species, and the same trade-offs between protection and opportunity costs apply. It follows that an integrated approach to the design of reserves is highly desirable [25], [26]. We explore such an integrated planning approach by combining our caribou optimization model with a model for coarse-filter conservation that we developed in a previous study [27].

The intent of our study is to provide land managers with efficient and effective reserve design options and a clear understanding of the economic trade-offs inherent in decisions concerning reserve design. Our hope is this will support the establishment of a reserve network that provides an optimal balance among competing conservation and economic objectives. More generally, we seek to advance the application of optimal resource allocation by extending the scope of its implementation. In addition to applying optimization in a single species metapopulation context, which is novel, we include economic tradeoffs and the projected effects of climate change in the optimization process, rather than focusing only on habitat representation.

## Methods

Our study area is comprised of Alberta's public lands (552,240 km^2^; Fig. 1). Polygons defining the range of each caribou herd were obtained from the Alberta Department of Sustainable Resource Development (Fig. 1). All current caribou range exists on public lands. Of the total range, 81% is allocated for industrial development (primarily forestry and oil and gas), 17% is protected within the provincial parks system, and 2% remains unallocated (Fig. 1).

### Model Inputs

The design of our reserves was based on five elements: (1) current industrial footprint, (2) current distribution of white-tailed deer, (3) projected effects of climate change, (4) opportunity cost, and (5) coarse-filter ecosystem representation.

We used the density of linear features (i.e., roads, pipelines, and seismic lines) as our indicator of the current industrial footprint. Linear features are closely linked to other anthropogenic features in our study area because seismic lines are needed in the early phase of oil and gas development and roads are required to access wellsites and forestry cutblocks. The density of linear features, expressed as km per km^2^ for each township, was derived from the Alberta Base Features dataset (Fig. S1).

We calculated the probability of the presence of white-tailed deer using a generalized linear model that included winter severity, length of the growing season, vegetation type, and total land-use footprint (Fig. S2). We selected this model from a suite of a priori climate, land-use, and habitat models on the basis of AIC value (w_i_ = 0.93). The presence/absence of white-tailed deer was based on snow track data collected between 2002 and 2009 from northern Alberta. Two datasets were used; one based on 9 km triangular transects, sampled on foot (n = 174), and the other based on 10 km linear transects, sampled on snowmobile (n = 125). The Integrated Landscape Management lab at University of Alberta and the Alberta Biodiversity Monitoring Institute conducted the sampling, selecting sites according to stratified random and systematic designs, respectively. Additional detail on the deer model is provided in Dawe [18].

Winter severity for the deer model was based on an index developed by DelGiudice et al. [22] that sums the number of days between November and April that the minimum temperature is below −17.7°C and snow depth is above 38 cm. We calculated the index annually using temperature and snow (water equivalent) data from Natural Resources Canada interpolated at a resolution of 100 km^2^ [28]. Growing season was also based on climate data from Natural Resources Canada, but was calculated at a resolution of 70 km^2^. Growing season started when the mean daily temperature was equal or greater than 5°C for at least five consecutive days, beginning on March 1, and ended when the minimum temperature reached −2°C after August 1. In our model we used the mean length of growing season from 1950–1999 and the mean winter severity index from 1961–2002. Vegetation type refers to the proportion of deciduous forest and proportion of wetland (excluding black spruce bogs) within a 500 m buffer on either side of the transect and was derived from the Alberta Ground Cover Classification [29]. Industrial footprint was the summed area of agricultural land, forest cutblocks and well pads within a 500 m buffer around each transect. Land-use data were derived from the Alberta Base Features dataset and the Alberta Vegetation Inventory.

The effect of climate change was based on the results of a previous study that used three bioclimatic envelope models to predict changes in the distribution of Alberta's ecosystems over the next 40 years [23]. Three general circulation models were used to derive the bioclimatic envelope models, representing a pessimistic hot/dry scenario (HAD-CM3-A1F1), an optimistic cool/moist scenario (PCM-B1) and a median scenario (CGCM2-B2). We used the projected distribution of parkland and grassland in 2050 (Fig. 1 in Schneider et al. [23]) as the input to our optimization model. We did this by combining the projections of all three bioclimatic envelope models into a single probabilistic estimate of the presence of parkland or grassland across our study area in 2050 (Fig. S3).

We defined opportunity cost as the value of foregone resource development opportunities resulting from a prohibition on new development within reserves. We expressed this variable as the net present value (NPV) of resources within new reserves as a proportion of the NPV of the total study area.

We determined NPVs for each of the four main industrial sectors active in our study — conventional natural gas, conventional oil, bitumen (a tar-like hydrocarbon found in oil sands), and forest products (Fig. S4) — using models developed by Hauer et al. [30]. These models projected expected resource flows, revenues and costs over time, and opportunity costs of capital in terms of discount or interest rates. From these projections we determined net resource values for each sector in present value terms (i.e., NPV). The true opportunity cost of establishing reserves is less than suggested by our estimates of NPV because industry is subject to various capacity constraints that limit the rate at which resources can actually be extracted. There are also opportunities for spatial substitution of activities. However, using these values in a relative fashion (i.e., expressing opportunity cost as a percent of total NPV) should be instructive for strategic planning.

For the oil and gas models the total amount of recoverable oil or gas available per geological layer in each section of land (∼278 ha) was derived from spatially explicit data on reserves and ultimate potential housed with the Alberta's Energy and Resources Conservation Board and the National Energy Board [31]. The flow of resources over time given successful drilling was derived from estimates published by the Alberta Department of Energy [32]. Seismic, operating costs, and capital costs were also obtained from the Alberta Department of Energy [32]. Drilling costs were derived from Petroleum Services Association of Canada [33]. For the capital intensive oil sands projects, costs and bitumen outputs per well were derived from the Alberta Department of Energy [34], [35]. For each section of land, flows of oil or gas were multiplied by forecasted oil and gas prices, derived from GLJ petroleum consultants Ltd. [36], [37]. This revenue stream was then discounted using a 4% real rate of return on investment. Discounted operating, drilling, and exploration costs were subtracted from this revenue to obtain the expected NPV for each land section.

The NPV of land under forest management accounts for less than 1% of total land resource values but was included for completeness. NPVs for forestry were obtained using the methods described in Hauer et al. [38]. The scheduling of forestry activities was based on maximizing NPV under provincial regulations including sustained yield constraints [38].

Our coarse-filter conservation scenario was based on an earlier study [27] and focused on representing all major ecosystem types within our study area. We defined ecosystems using the Natural Regions of Alberta map, which provides a hierarchical classification based on landform, soils, hydrology, climate, and dominant vegetation [39]. There are six Natural Regions and 21 Natural Subregions in the province and we used the Natural Subregions for our analysis (Fig. S5).

### Modeling Scenarios

We used Marxan to generate reserve designs that achieved our caribou protection targets while minimizing cost and/or demographic risk factors [12]. Marxan uses simulated annealing to identify the optimal configuration of planning units for a given conservation objective. Townships (∼9500 ha; Fig. S6) were used as the planning unit (n = 5784). Townships within the provincial protected area network were included in every design if 50% or more of the township was protected (Fig. 1). Townships that contained more than 50% private land were excluded from all designs (Fig. 1).

We investigated three scenarios. Scenario 1 included only the demographic risk factors (linear feature density, probability of deer, and probability of climate-induced habitat change). Our interest here was to visualize the optimal reserve configuration for the risk factors in the absence of cost constraints. The risk factors were investigated independently and in combination. We fixed the caribou protection target at 50% of the total provincial caribou range, which was best for illustrating patterns of selection and avoidance.

Scenario 2 included the three risk factors and opportunity cost. For these runs the three risk factors were combined, with equal weighting, into a single “risk” variable. Marxan was required to minimize both risk and cost as it worked to achieve a range of caribou protection targets (20, 40, 50, 60, and 80 percent of total provincial caribou range). Several weighting schemes were examined, including equal weighting of both risk and cost and alternatives in which either risk or cost was favoured. We compared the results in terms of spatial configuration and economic opportunity cost (i.e., the proportion of total NPV contained in the reserve system).

In Scenario 3 we added coarse-filter ecosystem representation to the model. To keep the overall number of model runs tractable we fixed the ecosystem target at 20%, which is the target identified in current Alberta land-use planning documents [40]. This means that reserve designs had to include a minimum of 20% of each Natural Subregion while also achieving the specified caribou protection targets and minimizing risk and cost (equal weighting).

For each scenario we ran Marxan 200 times and pooled the results. The repetitions were necessary because the simulated annealing algorithm used by Marxan to select the optimal configuration of planning units is inherently stochastic. Certainty is sacrificed to achieve search times that are practical (i.e., we accept solutions that are “very good” instead of trying to find the absolute “best”). The observed variance among solutions also helps to illustrate the relative importance of planning units by differentiating units that are always selected (core areas) from units that are only sometimes selected (flexible areas) and units that are never selected (areas of avoidance). We chose 200 repetitions because this was sufficient to generate stable mean NPV values, permitting meaningful comparisons to be made among scenarios. For visual display of reserve designs we calculated the probability of selection for each planning unit over the 200 repetitions of a scenario run and linked this to a map of Alberta townships.

## Results

**Figure 3:**
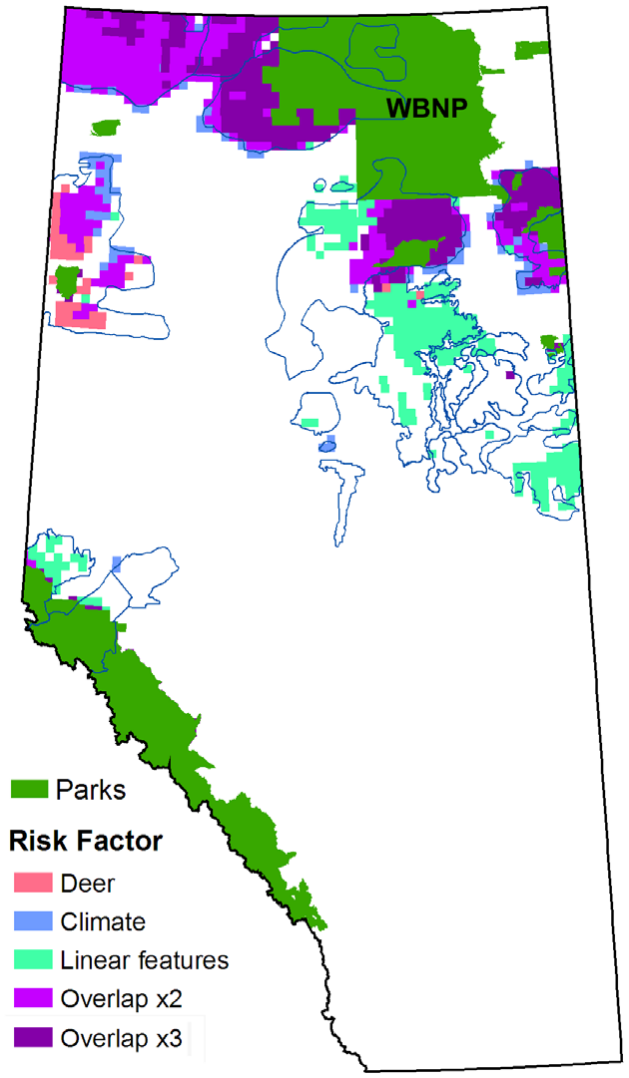
Planning units selected in Scenario 1. The map represents a composite of three model runs, one for each of the demographic risk factors. The caribou protection target was 50% in all cases. Units that were selected by more than one model are labelled as “Overlap”. For clarity, only planning units with a high probability of selection (>50%) are shown.

There was a high degree of spatial overlap in the optimized reserve designs for the three individual demographic risk factors with no cost constraint (Scenario 1; Fig. 3). All three designs exhibited a strong preference for caribou range adjacent to Wood Buffalo National Park and an avoidance of caribou range in east-central Alberta, where the main oil sands deposits are located (Fig. 3).

**Figure 4:**
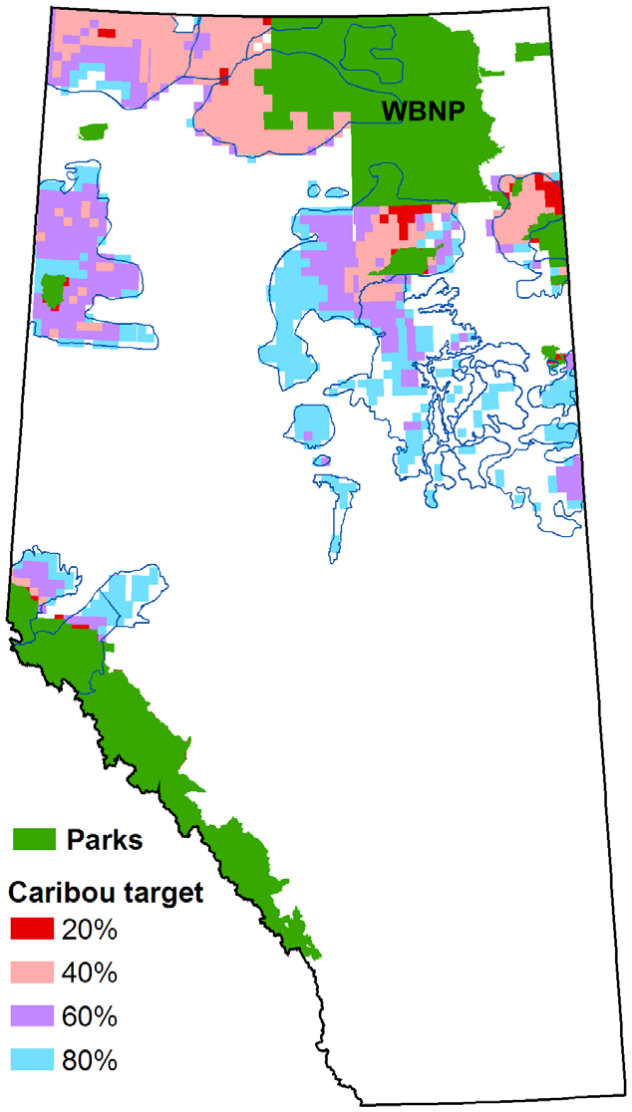
Planning units selected in Scenario 2. Results are shown for four levels of caribou protection target. All models included risk and cost. For clarity, only planning units with a high probability of selection (>50%) are shown. Note that most of the units selected at the 20% target lie within existing protected areas and so are hidden from view.

**Figure 5:**
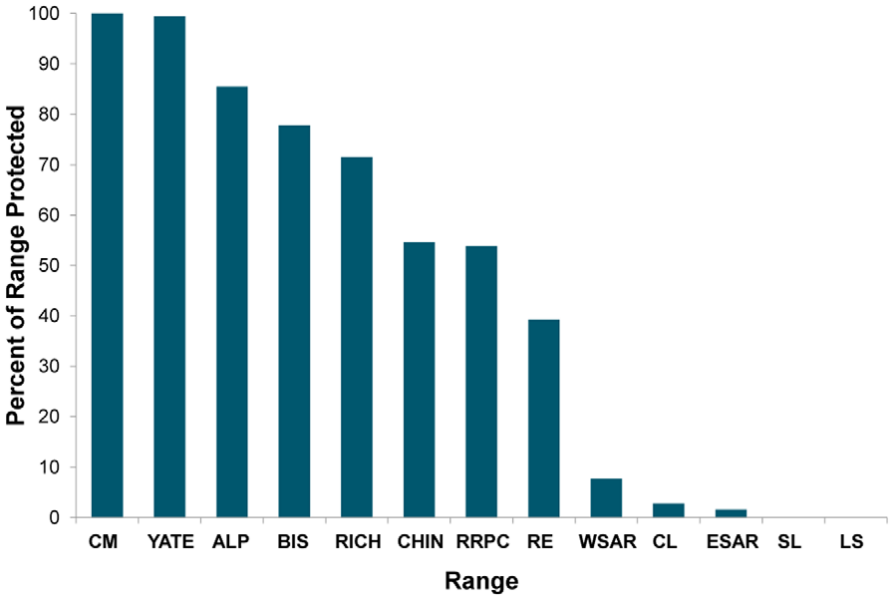
Percentage of each range protected in Scenario 2. The model included risk and cost and the overall caribou protection target was 50%.

The same general pattern of selection and avoidance was evident when the model contained all demographic risk factors and opportunity cost (Scenario 2; Fig. 4). When the caribou protection target was less than 50%, most of the range selected by the model was adjacent to Wood Buffalo National Park and along Alberta's northern boundary. Caribou range in east-central Alberta (the oil sands region) was generally avoided, even when the caribou protection target was 80%. These spatial patterns of selection resulted in large differences in the percentage of each range that was protected (Fig. 5).

**Figure 6:**
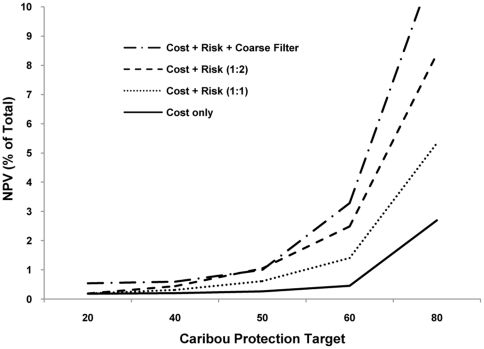
Opportunity cost of the reserve system relative to the caribou protection target. Cost is expressed as a percentage of the NPV of the entire study area. Three variations of Scenario 2 are shown, illustrating different relative weightings of cost to risk. Results are also shown for Scenario 3 (coarse-filter).

The opportunity cost of protection in Scenario 2 was less than 1% of the total NPV of the study area until the caribou target reached 50% (Fig. 6). Opportunity cost rose rapidly once the caribou target exceeded 60%. Increasing the relative weighting of risk to cost in the model resulted in higher opportunity costs (Fig. 6). However, differences among the models were minor until the caribou target exceeded 50%.

**Figure 7:**
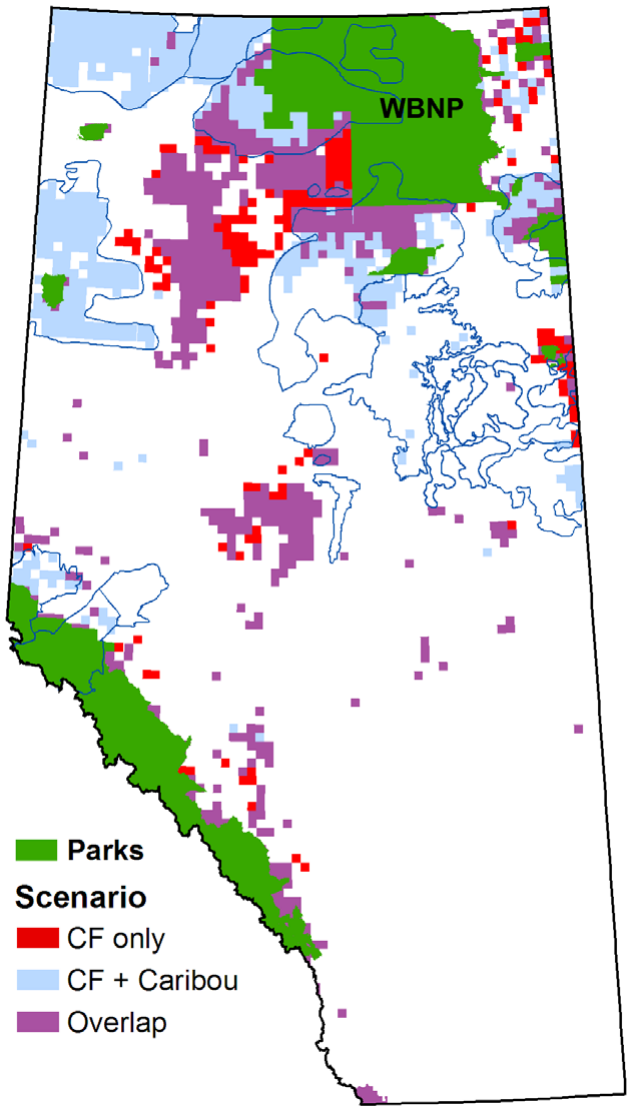
Planning units selected in Scenario 3. Units labelled CF (red) were selected in a model that included only coarse-filter targets (at 20%). Units in blue were selected in a model that also included a caribou protection target of 60%. Units labelled “Overlap” (purple) were selected by both models.

The addition of coarse-filter ecosystem representation to the model (Scenario 3) resulted in the highest opportunity costs of our study. However, opportunity cost still remained below 1% of the total NPV of the study area until the caribou target reached 50% (Fig. 6). Optimized reserve designs for Scenario 3 indicate that planning units selected for the protection of caribou range help to achieve coarse-filter ecosystem representation targets (Fig. 7; planning units in red are no longer required when a caribou target of 60% is added to the model). This offset potential is exhausted, however, once the caribou target is greater than 50%. Beyond this point most of the planning units selected for caribou are additive to the basic coarse-filter design.

## Discussion

Given the incompatibility between industrial development and caribou viability [2], [5], [15], [16], habitat protection represents a key strategy for caribou conservation. In a resource-rich province such as Alberta, however, the opportunity cost of protection presents a serious barrier to the establishment of new reserves. Indeed, there is no mention of habitat protection in the Alberta Caribou Recovery Plan [41].

Our findings suggest that the prospects for habitat protection may be greater than previously supposed. By optimizing the design of the reserve system, 60% of current caribou range can be protected (including 17% in existing parks) while maintaining access to over 98% of the value of resources on Alberta's public lands. One of the main reasons for this favourable outcome is that the distribution of resource values is highly variable across our study area, largely because of the presence of the oil sands deposits. Optimization techniques are particularly effective in minimizing the cost of conservation solutions when variance of the cost layer is high [9], [24], [42]. In practical terms, by avoiding protection within the oil sands region, sufficient economic returns are generated to the province to adequately offset the opportunity costs of protection in other areas.

Our composite map of optimized reserve designs across a range of protection targets (Fig. 4) illustrates the spatial prioritization of protection options. The planning units with the highest priority for protection are consistently found around the periphery of Wood Buffalo National Park and along Alberta's northern border. The planning units with the lowest priority for protection are found in east-central Alberta, where Alberta's oil sands deposits are centered. In part, this pattern reflects the model's avoidance of townships with high resource value. But equal weighting was given to the avoidance of demographic risk factors; therefore, the observed pattern represents a compromise solution. The amount of compromise actually needed was minimal because the spatial distribution of risk and cost turned out to be very similar. This is fortunate because it means that land managers are not forced to choose between minimizing cost or risk when selecting reserves.

A certain amount of concordance in the distribution of cost and risk was expected, in that our composite measure of risk included industrial footprint (density of linear features), which tends to be concentrated in areas where resource values are greatest. But risk also included the presence of deer, which is only partially related to industrial development. Dawe [18] found that the most important factor in the recent northward expansion of deer range in Alberta is climatic warming. Finally, our modeling of risk also included the effects of climate change on the future availability of suitable caribou habitat, and this has no relationship to industrial development at all.

A case in point is the oil sands region, which is obviously avoided on the basis of high opportunity costs. It turns out this region is also the least desirable from the perspective of combined demographic risk, as illustrated by the risk-only model runs of Scenario 1 (Fig. 3). Ironically, the only risk model to utilize the oil sands region to any extent was the linear feature model (Fig. 3). The behaviour of the linear feature model reflects the fact that oil sands development has only recently become economically viable and so substantial parts of the region are still relatively intact (Fig. S1). The deer-only and climate-only models completely avoided the oil sands region.

The proportion of each range that was protected under the optimized reserve design varied widely among herds. For example, an overall protection target of 50% resulted in 100% protection of CM and YATE and less than 5% protection of LS, SL, and some of the ranges in the oil sands region. It is notable is that the herds with the lowest priority for protection under the optimization approach include the herds that are the primary focus of current provincial recovery efforts, based on their high risk of extirpation (see management implications below).

Our coarse-filter scenario demonstrated that conservation gains can be achieved through integrated conservation planning. By preferentially selecting planning units that achieved both caribou targets and ecosystem representation targets, the optimization model was able to minimize the incremental cost of adding coarse-filter conservation objectives to the design. There was a limit to the benefits of integration, however, because caribou habitat is relatively uniform in terms of ecosystem composition. Once these ecosystem types were adequately represented, further protection of caribou habitat was additive to the design. This occurred once the caribou target exceeded 50%.

As with any modeling study, our findings must be considered in light of underlying assumptions and limitations. One concern is that our analysis of the economic implications of protection did not include potential benefits. Though it is clear that reserves provide societal benefits beyond the conservation of biodiversity, estimating of the equivalent dollar value of these benefits and their distribution across space was beyond the scope of this study. If these benefits were accounted for the net opportunity cost of protection would be lower than reported here [43]. Furthermore, the establishment of new reserves does not imply the simple idling of industrial capacity, but a reallocation to other parts of the landscape. This also serves to reduce real opportunity costs. The implication is that our findings regarding the trade-offs between economic opportunity costs and conservation objectives represent a worst-case scenario (in terms of cost).

Although our model included what are arguably the most important risk factors for caribou in Alberta, our list was not exhaustive. For example, we did not include the potential spread of chronic wasting disease from deer to caribou or stochastic demographic risk associated with small population size [44]. Furthermore, the population effects of the factors we included have only been described qualitatively. Likewise, our estimates of NPV, however well grounded by government data, may not be predictive of opportunity costs in the future because of unforeseen events. For example, timber resources may be lost through fire or technological advances might raise the value of petroleum resources that cannot be profitably extracted at present. In light of these limitations, the risks and costs used in our study should not be considered accurate projections of the future but elements of plausible and meaningful modeling scenarios that are useful in the context of strategic decision making.

Another limitation of our study is that our conservation designs were not comprehensive. For example, we did not consider factors affecting metapopulation dynamics, such as the number of reserve replicates, minimum reserve size, or contiguity of planning units (though the amount of natural contiguity was actually quite high). Therefore, while our results provide useful guidance regarding relative spatial priorities for protection, they should not be considered adequate for the delineation of reserve boundaries.

We also did not include genetics in our modeling. This raises the concern that the mountain ecotype, represented by just three herds in west-central Alberta adjacent to the Rocky Mountains, may be underrepresented relative to the larger boreal ecotype comprising the remaining herds [45]. In fact, representation was well balanced between the two ecotypes, with a slight overrepresentation of the mountain region on a proportional basis. In practical terms, if a decision is made to protect habitat within the mountain region on the basis of genetic concerns, land managers can use our township-scale map of relative priorities (Fig. 4) to identify the best prospects for protection within the region.

### Management Implications

Recovery efforts to date have been focused on the herds at greatest risk of extirpation, which happen (not coincidentally) to occupy ranges with high resource value. With this approach, the potential for protection is much less than suggested by our findings. For example, the opportunity cost of protecting the CL range (the herd with the fastest rate of population decline) is 8.2 times the cost of the entire optimized reserve design with a 60% protection target.

The upshot is that, in the face of capacity limitations, allocation based on the risk of extirpation achieves relatively little protection and fosters the perception that protection is too expensive to be seriously considered. Moreover, by targeting the weakest herds the reserves are located where the probability of success is lowest. This approach seems ill advised given that all but one of Alberta's caribou herds are in decline and require attention [6]. There is a strong parallel here to multi-species systems, where it has been shown that allocating limited resources solely to the most endangered species will typically not minimise the number of extinctions in the long-term because it does not account for the risk of less endangered species going extinct in the future [46].

We conclude that the prospects for caribou recovery would be improved if the allocation of available conservation capacity was based on maximizing long-term outcomes at the provincial scale rather than dwelling on the fate of individual herds [7], [11], [46]. This implies a shift in mindset from avoiding short-term failure to ensuring long-term success and means that factors such as economic trade-offs and climate change need a much higher profile in the planning process than they have had in the past. As for the amount of habitat to be protected, this is a matter of balancing conflicting societal objectives – there is no objective “right” number. Our opportunity cost curve (Fig. 6) illustrates the trade-offs involved and can provide guidance to decision makers in this respect.

## Supporting Information

Figure S1 **Density of linear features, by township.**
(TIF)

Figure S2 **Probability of the presence of white-tailed deer, by township.**
(TIF)

Figure S3 **Projected distribution of parkland and grassland bioclimatic zones in 2050.** The map presents the overlaid projections for these two zones from three climate models.
(TIF)

Figure S4 **Net present value of petroleum and forestry resources, by township.**
(TIF)

Figure S5 **The Natural Subregions of Alberta.** Note that grassland and parkland subregions were largely excluded from the analysis because they contain little public land (see Fig. 1).
(TIF)

Figure S6 **Planning units used in the Marxan modeling.** Private land is excluded from all designs (locked-out) and existing protected areas are included in all designs (locked-in).
(TIF)
