# Supplementary figures and images for: Selection of Reserves for Woodland Caribou Using an Optimization Approach
Source: PLoS One. 2012 Feb 20;7(2):e31672. doi: 10.1371/journal.pone.0031672 (PMC3282734; doi:10.1371/journal.pone.0031672)

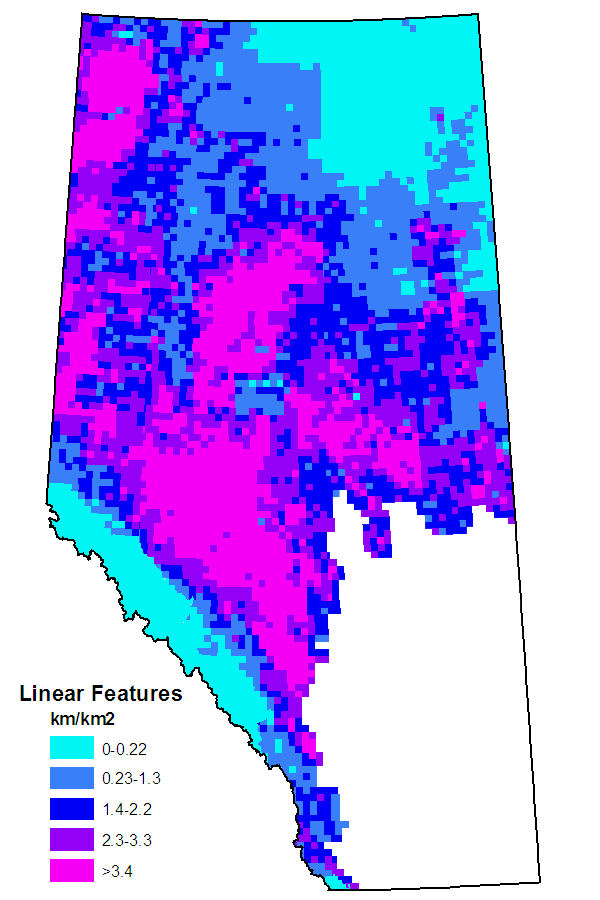

Supplement: Figure S1 — Density of linear features, by township. (TIF) [file pone.0031672.s001.tif]

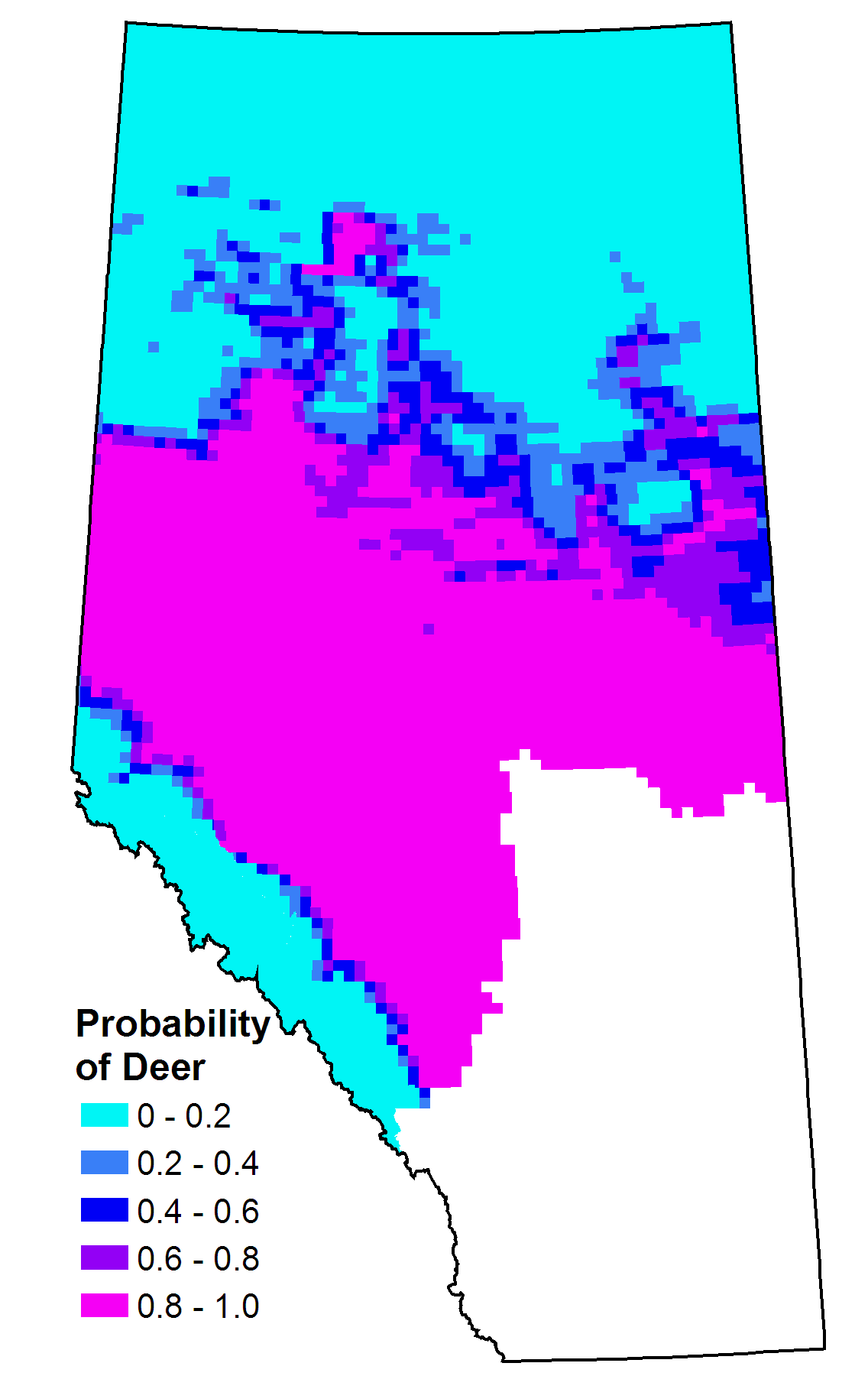

Supplement: Figure S2 — Probability of the presence of white-tailed deer, by township. (TIF) [file pone.0031672.s002.tif]

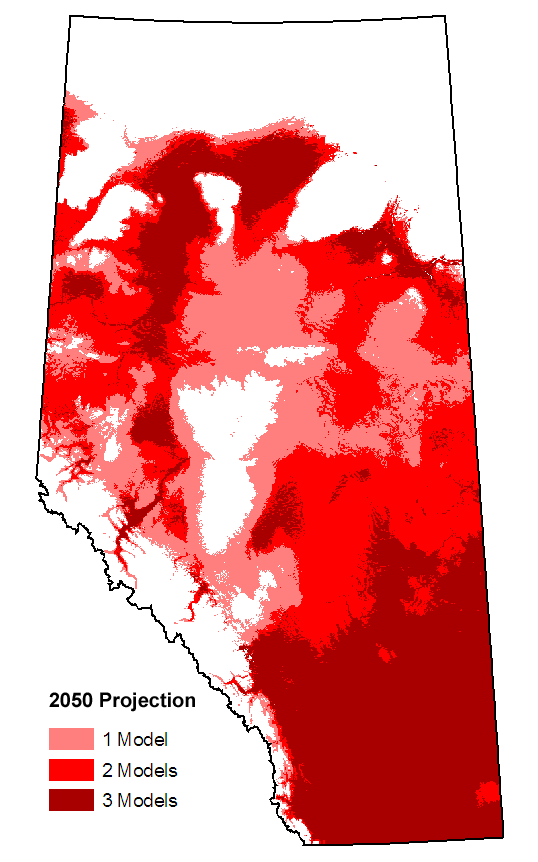

Supplement: Figure S3 — Projected distribution of parkland and grassland bioclimatic zones in 2050. The map presents the overlaid projections for these two zones from three climate models. (TIF) [file pone.0031672.s003.tif]

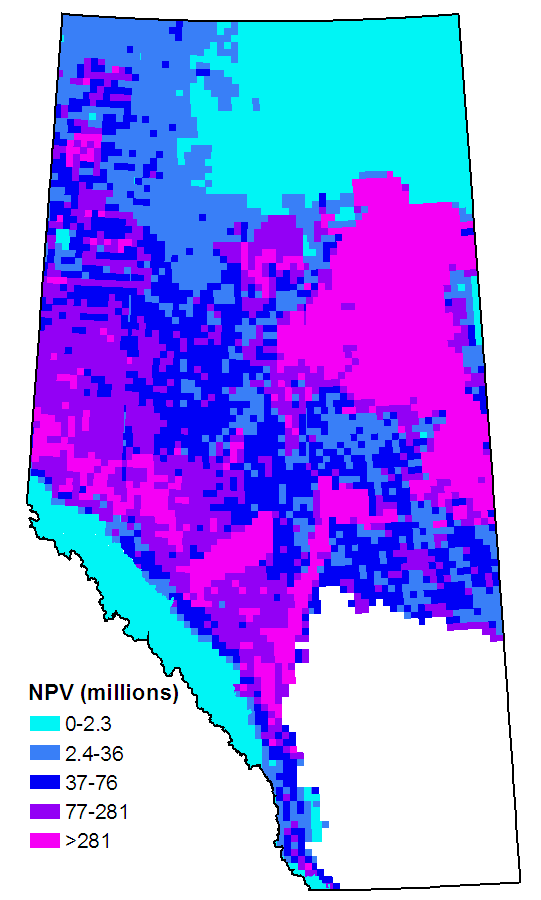

Supplement: Figure S4 — Net present value of petroleum and forestry resources, by township. (TIF) [file pone.0031672.s004.tif]

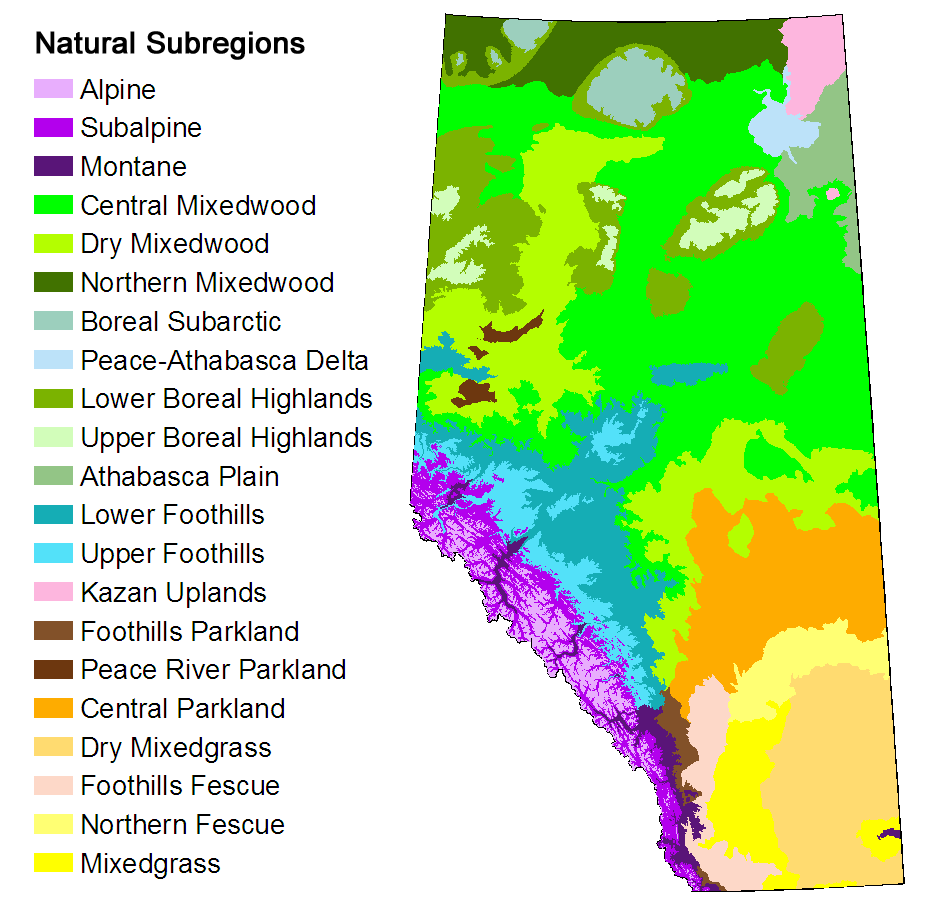

Supplement: Figure S5 — The Natural Subregions of Alberta. Note that grassland and parkland subregions were largely excluded from the analysis because they contain little public land (see Fig. 1). (TIF) [file pone.0031672.s005.tif]

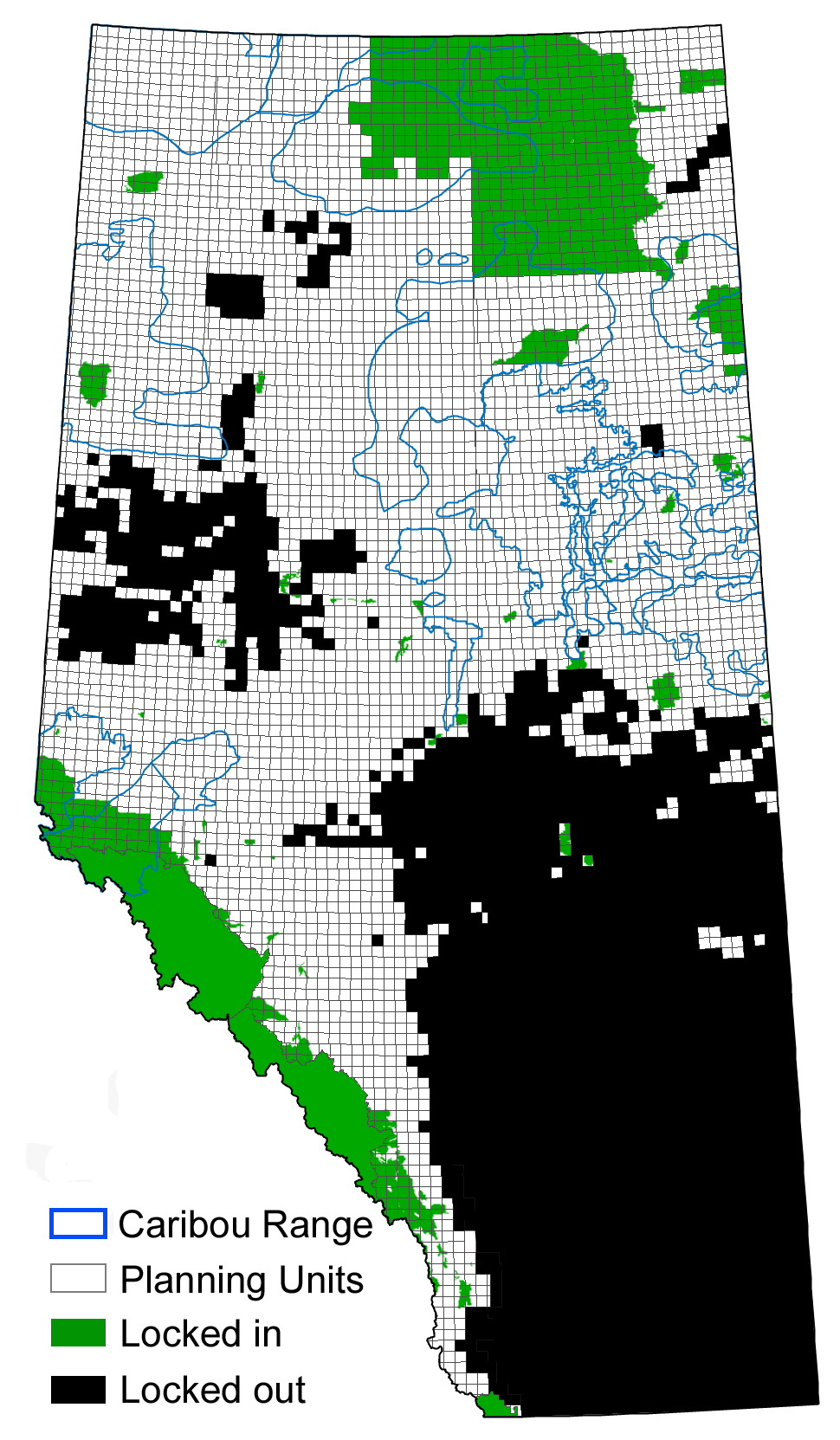

Supplement: Figure S6 — Planning units used in the Marxan modeling. Private land is excluded from all designs (locked-out) and existing protected areas are included in all designs (locked-in). (TIF) [file pone.0031672.s006.tif]
